# Radiation damage and derivatization in macromolecular crystallography: a structure factor’s perspective

**DOI:** 10.1107/S2059798315021555

**Published:** 2016-03-01

**Authors:** Robin L. Owen, Darren A. Sherrell

**Affiliations:** a Diamond Light Source, Harwell Science and Innovation Campus, Didcot, Oxfordshire OX11 0DE, England

**Keywords:** phasing, radiation damage, macromolecular crystallography

## Abstract

During, or even after, data collection the presence and effects of radiation damage in macromolecular crystallography may not always be immediately obvious. Despite this, radiation damage is almost always present, with site-specific damage occurring on very short time (dose) scales well before global damage becomes apparent. A result of both site-specific radiation damage and derivatization is a change in the relative intensity of reflections. The size and approximate rate of onset of X-ray-induced transformations is compared with the changes expected from derivatization, and strategies for minimizing radiation damage are discussed.

## Abstract 2

Both site-specific radiation damage and heavy-atom derivatization result in small changes to the intensity of reflections. The size of the change owing to each is calculated and compared for individual reflections.

## 1. Introduction   

Radiation damage in macromolecular crystallography (MX) is an inevitable and, for the most part, unwelcome aspect of data collection. In many cases the presence of radiation damage is made clear by changes in metrics such as the diffracting power: a common observation during data collection at synchrotron sources is a decrease in the diffracting power of a crystal as the collection of a data set progresses. The decay in diffracting power is particularly striking at higher resolution, with spot patterns in outer resolution shells often fading to become invisible by the end of data collection. Changes in the crystal mosaicity, unit-cell volume or *B* factor may be less immediately visible, but nonetheless retrospective analysis can also reveal the heavy footprint left by X-rays.

Dramatic changes in diffracting power can be mitigated by reducing the exposure time or attenuating the X-ray beam. This strategy, however, gives rise to additional challenges: even if a change in the diffracting power, or another easy-to-follow metric, is not observed, radiation damage is still occurring. Site-specific damage occurs much faster than global damage and can be much more difficult to track or quantify. Complementary methods such as UV–Vis or fluorescence spectroscopy provide a means of confirming the onset of localized damage, and the reduced dose scales associated with site-specific damage have been quantified using a range of complementary methods. These include disordering of selenomethionine side chains (2 MGy; Holton, 2007 ▸), reduction of copper nitrate reductase (1.5 MGy; Hough *et al.*, 2008 ▸), cleavage of anomalous scatterers (0.5 MGy; Oliéric *et al.*, 2007 ▸), photoreduction of putidaredoxin (0.3 MGy; Corbett *et al.*, 2007 ▸), reduction of myoglobin by X-rays (0.045 MGy; Owen *et al.*, 2011 ▸) and γ-radiolysis (0.020 MGy; Denisov *et al.*, 2007 ▸).

The insidious nature, or indeed apparent absence of, site-specific damage poses a serious problem as it may be unclear whether X-ray-induced artefacts are present in the derived electron-density map or why the experiment has failed. Here, we will briefly review the different forms of radiation damage and the dose scales on which these occur, and illustrate the effect that these can have on the structure factors observed during the experiment. Calculation of structure factors will show the changes owing to radiation damage to be comparable to, and in some cases greater than, the changes expected from derivatization.

## 2. Damage is proportional to dose   

This article is not intended to be a comprehensive review of radiation damage in MX: for this, the reader is referred to recent excellent reviews such as Holton (2009 ▸) and Garman (2010 ▸). A fundamental observation is that at cryotemperatures damage is proportional to the energy per unit mass, or dose, absorbed by a crystal. At temperatures above ∼200 K, experiments have shown that damage can be, at least partially, outrun (Warkentin *et al.*, 2013 ▸; Owen *et al.*, 2014 ▸). Absorbed dose is still however the dominating factor: all crystals have a finite life dose in the X-ray beam. Below, we will primarily consider the dose scales and limits associated with cryo-crystallography.

The dose absorbed by a crystal during an experiment can be calculated using a program such as *RADDOSE* (Zeldin *et al.*, 2013 ▸; Paithankar *et al.*, 2009 ▸). Absorbed dose is measured in grays (Gy). The most convenient unit for macromolecular crystallographers is the megagray (MGy), as typically doses of this order are achieved in an experiment at a synchrotron beamline (Table 1 ▸). Table 1 ▸ shows that doses of the order of tenths of a megagray can be easily reached with the beam sizes readily achievable at many beamlines. This is irrespective of the protein of interest: the X-ray cross-section of most proteins is the same to within a factor of two, and the dose absorbed by a crystal is dominated by the beam parameters. Moving to a microfocus beamline with a similar flux, but a greatly increased flux density, results in an order-of-magnitude increase in the absorbed dose and hence an order-of-magnitude decrease in the crystal lifetime.

**Figure 1:**
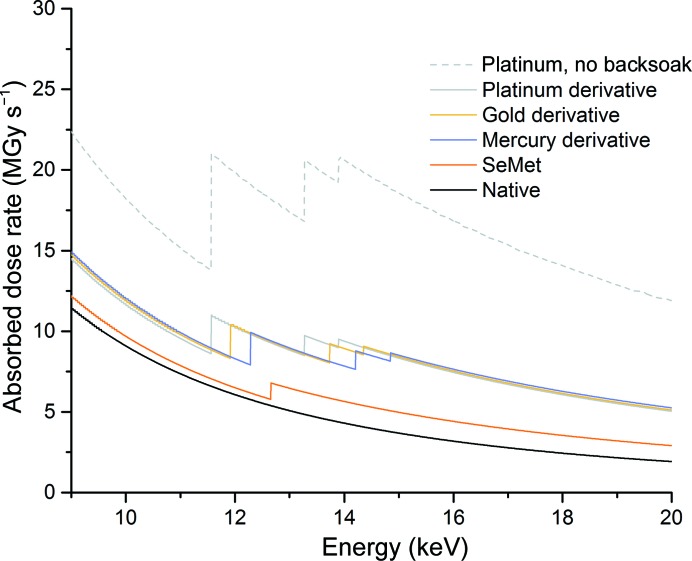
Change in absorbed dose as a function of energy for native and derivatived FcγRIII crystals. Dose calculations assume a beam size of 10 × 10 µm and a constant incident flux of 1.5 × 10^12^ photons s^−1^. For all derivative dose calculations two heavy atoms were added to each monomer of 175 amino acids. FcγRIII contains no methionines, but the absorbed dose for a selenomethionine derivative was calculated for illustrative purposes using the average frequency of methionine (1.8%; three per monomer). For the non-backsoaked platinum derivative a K_2_PtCl_4_ concentration of 250 m*M* was used.

A particularly inconvenient factor for a phasing experiment is that heavy atoms have a disproportionally large effect on the absorbed dose; this is illustrated in Table 2 ▸ and Fig. 1 ▸. As an experiment is typically set up in terms of elapsed time (and attenuation), the dose per second is shown as a function of energy in Fig. 1 ▸, but it should be noted that while increasing the energy appears to be an efficient way of reducing the absorbed dose, the elastic scattering cross-section also decreases as a function of energy. Thus, increasing the energy of the incident X-rays reduces the absorbed dose per second but an increased exposure time is required to record a useful diffraction pattern. Instrumentation such as detectors may also be optimized for operation around 12 keV, so this should also be considered. Table 2 ▸ and Fig. 1 ▸ show that the addition of a relatively small number of heavy atoms (*Z* greater than or equal to that of sulfur) can more than double the absorbed dose, with the effect being most pronounced at elemental absorption edges. The effect of heavy-atom absorption is greatly exacerbated in experiments in which the heavy atom is soaked into the crystal. Disordered heavy atoms present in the solvent surrounding the protein do not contribute to any observed anomalous scattering, but contribute greatly to the dose absorbed by a crystal. The deleterious effect of this is illustrated by the contrast in absorbed dose between two platinum derivatives. If a crystal is perfectly backsoaked, such that the only platinum remaining is that which is covalently bound, then the absorbed dose increases by a factor of 1.6 compared with the native just above the Pt *L*_III_ edge. If no backsoaking is performed then the absorbed dose increases by a factor of 3.1 compared with the native at the same energy.

Knowledge of the crystal contents and beam parameters can thus allow the dose absorbed by a crystal during an experiment to be calculated. Comparison of this dose with systematic studies allows the degree of global and site-specific damage to be estimated and can provide a guide for the tolerable dose, which may change according to the primary aim of the experiment.

## 3. Dose scales for damage   

One of the most familiar symptoms of radiation damage is the fading of diffraction patterns, especially at high resolution. Global effects such as these occur on the order of tens of megagrays, with Howells *et al.* (2009 ▸) suggesting a criterion of 10 MGy per Å resolution based on a number of studies in the literature. Thus, when collecting a data set to 3 Å resolution a dose of ∼30 MGy can be tolerated, while for a 1 Å resolution data set this reduces to ∼10 MGy. When compared with the doses in Tables 1 ▸ and 2 ▸, these dose limits appear to be rather generous, with total exposure times of several (tens) of seconds possible before they are reached. As outlined in §[Sec sec1]1, the dose scales associated with site-specific damage are significantly less than this however, with damage observed on dose scales of tens of kilograys in some cases.

It is clear when comparing site-specific limits, and the dose rates in Tables 1 ▸ and 2 ▸, that an experimenter can easily exceed any of these during data collection. On some beamlines doses of the order tens of kilograys are achievable within the first few images of data collection, meaning that the vast majority of data collected may be representative of a damaged or intermediate structure. Extreme caution must be exercised in the interpretation of electron-density maps, especially at active sites or metal centres. In the following sections we aim to illustrate how site-specific damage and non-isomorphism affect the intensity of reflections, and compare the size of changes with those expected from heavy-atom derivatization.

## 4. Quantifying changes owing to radiation damage and derivatization   

In the examples described here, the soluble protein FcγRIII is used as a test case (Zhang *et al.*, 2000 ▸). The structure of FcγRIII has been deposited in the PDB with accession code 1fnl, with unit-cell parameters *a* = 67, *b* = 86, *c* = 36 Å in space group *P*2_1_2_1_2. Each FcγRIII monomer consists of 175 amino acids, with no methionines and four cysteines. The four cysteine residues form two disulfide bridges. The unit cell contains approximately 8000 atoms, of which 16 are sulfur.

Structure factors can be written as the sum of scattering from all atoms in the unit cell. For the structure factor *F*(*hkl*) this is most clearly illustrated when written as
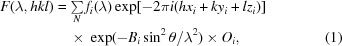
where *N* is the number of atoms in the unit cell, the atomic scattering factor *f_i_* is the scattering by the *i*th atom and the second, third and fourth terms in the sum are the phase, Debye–Waller factor and occupancy, respectively. At low resolution, the non-energy-dependent part of the atomic scattering factor, *f*_0,*i*_, is equal to the number of electrons in the atom, but this reduces at higher resolution according to the shape of the electron distribution around the atom. *O_i_* is the occupancy of the *i*th atom. The phase factor exp[−2π*i*(*hx* + *ky* + *lz*)] represents the phase shift of the scattered wave relative to that of the incident wave. The Debye–Waller factor exp(−*B*sin^2^θ/λ^2^) smears the electron density, representing thermal and crystal disorder: the quantity *B* represents the breadth of the smearing.

**Figure 2:**
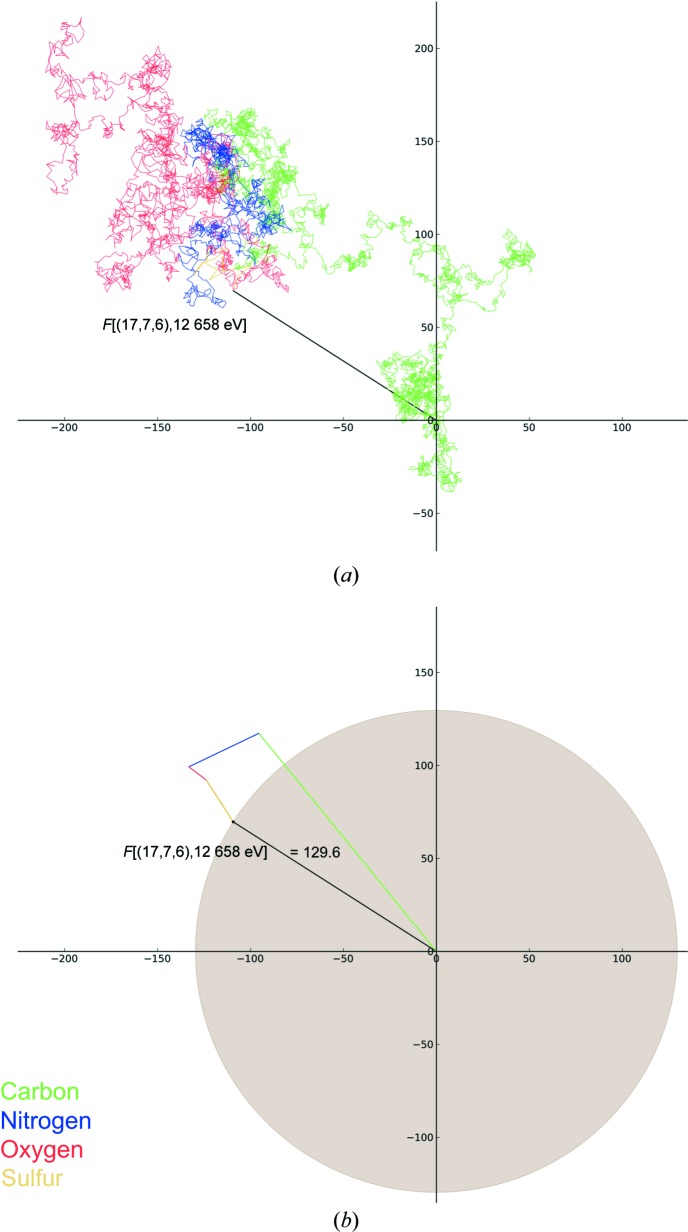
Argand diagram showing the contribution of all atoms to reflection (17, 7, 6) in FcγRIII (*a*). The plot can be simplified by grouping the contribution of each element (*b*). In both cases the contribution of each atom is colour-coded according to element type. In this and in subsequent figures the amplitude of the structure factor (129.6) is shown and is equal to the radius of the shaded circle. Percentage changes given in figures or in the text refer to changes in intensity (proportional to the area of the shaded circle).

Using the program *DeskArgand* (D. Sherrell, unpublished work) all terms in the summation shown in (1) can be plotted in the complex plane, illustrating the contribution of each atom in the unit cell to a particular structure factor (Fig. 2 ▸). Energy-independent scattering is calculated using Cromer–Mann coefficients (Cromer & Mann, 1968 ▸) and the *d*-spacing of the diffraction plane. Anomalous dispersion is calculated using Cromer–Liberman values (Cromer & Liberman, 1970 ▸) for all atoms. The structure-factor calculation takes into account occupancy and the Debye–Waller factor, but it does not include the Lorentz factor or polarization.

Starting at the origin, the contribution of each atom is added nose-to-tail in turn, with the resulting vector sum representing the amplitude and phase of the reflection. This vector sum is shown in Fig. 2 ▸(*a*) for reflection (17, 7, 6), a reflection at a resolution of 3.2 Å. This is a randomly selected nonzero (or close to zero) reflection chosen to clearly illustrate how the many terms in (1) sum to result in the observed structure factor *F*. This Argand diagram illustrates several aspects of how the amplitude and phase of a reflection are determined. Most obvious is the random walk taken: the amplitude of *F*(*hkl*) is much less than the linear sum of the atomic scattering factors in the unit cell. The dominance of the phase factor in (1) can be seen and even for a low-resolution reflection the contribution of a particular atom to *F*(*hkl*) may be much less than *f_i_* depending on the phase angle. Fig. 2 ▸(*b*) simplifies the Argand diagram for reflection (17, 7, 6), with the contribution of atoms to the final structure factor grouped by element type.

## 5. How radiation damage affects structure factors   

Certain amino acids such as glutamate, aspartate and tyrosine are particularly susceptible to localized damage, but one of the most well recognized calling cards that X-rays leave in an electron-density map is changes at disufide bonds (Burmeister, 2000 ▸; Weik *et al.*, 2000 ▸; Ravelli & McSweeney, 2000 ▸). The rapid formation of large numbers of solvated electrons upon X-ray irradiation, and the ability of these electrons to travel through a crystal, provides a means for the rapid cleavage of disulfide bonds even at 100 K (Sutton *et al.*, 2013 ▸).

**Figure 3:**
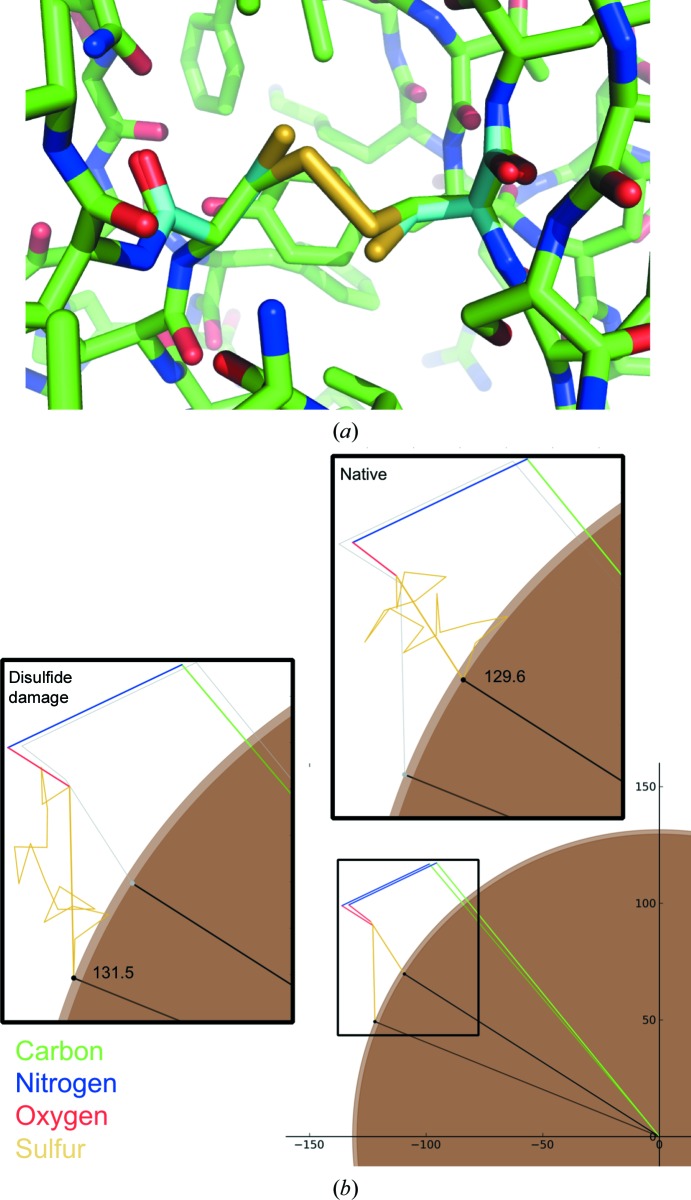
Structural change made when stretching the disulfide bond Cys110–Cys154 from 2.04 to 3.22 Å (*a*). For clarity, C atoms from the model with the stretched disulfide are shown in cyan. (*b*) shows the change in the structure factor (17, 7, 6) resulting from the disulfide stretch shown in (*a*). The structure factor calculated using the deposited coordinates is highlighted in the upper right inset and that from the stretched disulfide in the left inset.

In order to assess the impact of disulfide-bond cleavage on reflection (17, 7, 6), each of the disulfide bonds present in FcγRIII was stretched. The bond Cys29–Cys71 was increased from 2.06 to 3.17 Å, while Cys110–Cys154 was increased from 2.04 to 3.22 Å; the stretch of the latter is shown in Fig. 3 ▸(*a*). The structure factor (17, 7, 6) was then recalculated and plotted in the complex plane (Fig. 3*b* ▸). Despite the relatively small number of atoms involved (the 16 S atoms involved comprise ∼0.2% of the total number of non-H atoms present in the unit cell) and the small changes made, there is a clear effect on both the amplitude and phase of the structure factor. In this case the intensity increases by 3% when the disulfide bonds are stretched. It was noted some time ago that specific structural damage causes the intensity of individual reflections to increase or decrease (Blake & Phillips, 1962 ▸); the example shown in Fig. 3 ▸ illustrates exactly how small movements of a subset of atoms result in an increase in intensity as radiation damage progresses.

**Figure 4:**
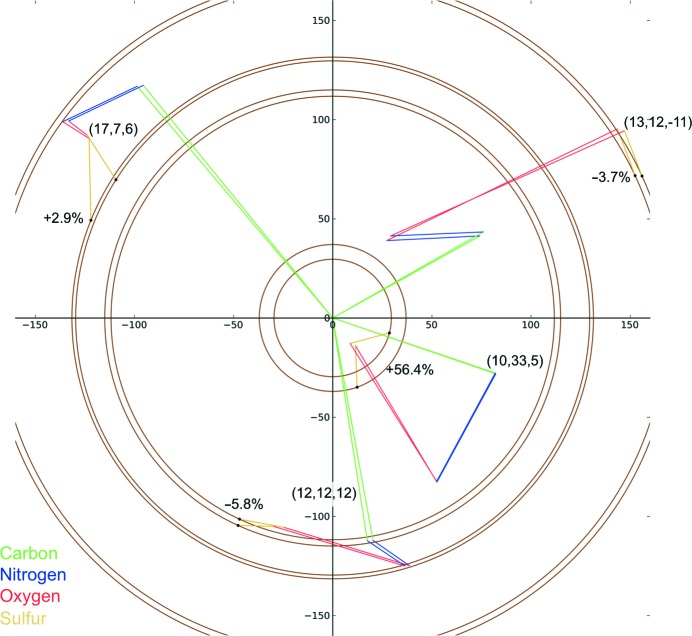
Change in a set of four reflections when both disulfide bonds in FcγRIII are stretched. The *hkl* and the change in intensity of each reflection are shown. The reflections are at resolutions of 3.2 Å (17, 7, 6), 2.6 Å (13, 12, −11), 2.5 Å (12, 12, 12) and 2.3 Å (10, 35, 5). The four reflections were chosen randomly, although the random choice was repeated until a nonzero, visually non-overlapping set of four was selected.

Site-specific changes affect different reflections by different amounts. This is illustrated for four different reflections in Fig. 4 ▸. In each case the same change to the disulfide bonds in FcγRIII has been made; however, the change in intensity varies from −5.8 to +56.4% between reflections. Thus even a small structural change, which can occur at low doses, can result in significant changes to individual reflections. Such changes may occur well before a significant decay in the global diffracting power is observed. In order to quantify the effect of site-specific damage on diffracting power and characterize overall trends, the calculation described in §[Sec sec4]4 was repeated for a set of 10 000 reflections. The change in total diffracting power owing to site-specific damage alone was, as might be expected, extremely small, with a calculated reduction of 0.25%; the median change in the intensity of a reflection was 16.1%.

**Figure 5:**
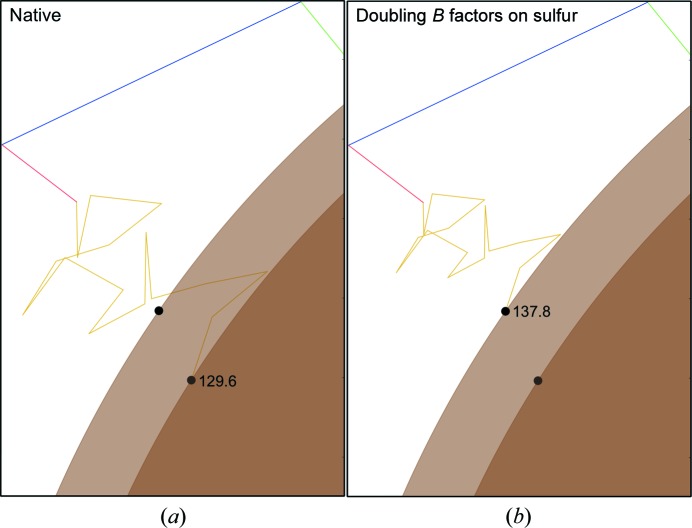
Change in structure factor when the *B* factor is selectively increased. (*a*) shows the structure factor calculated using the deposited *B* factors of FcγRIII, while in (*b*) all sulfur *B* factors have been doubled. The result of this change is that the intensity of the reflection increases by 13.1%.

The effects of the Debye–Waller term in (1) on the structure factor can also be illustrated in the complex plane. A change in the Debye–Waller factor may result from radiation damage if damage causes S atoms in different asymmetric units/unit cells to have different positions as site-specific changes occur at slightly different rates across the crystal. This heterogeneity is represented by a differential change in *B* factor for different groups of atoms. To illustrate the effect of this on a single structure factor, the *B* factor of the S atoms forming disulfide bonds in FcγRIII was doubled and the structure factor was recalculated; the resulting changes are shown in Fig. 5 ▸. Selectively increasing the *B* factor of a subset of atoms reduces the contribution of those atoms to the structure factor. For this particular reflection, doubling the *B* factor of S atoms again increases the observed intensity, this time by 13.1%. The median change in the intensity of a reflection is 8.7% (based on a set of 10 000 reflections).

## 6. How derivatization affects structure factors   

It is well known that the addition of a small number of heavy atoms to a protein results in changes in the intensity of reflections. A simple rule of thumb for the size of the change 〈Δ*I*〉/*I* induced is
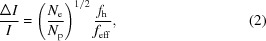
where *N*_e_ is number of heavy atoms, *N*_p_ is the number of non-H protein atoms, *f*_h_ is the atomic number of the heavy atom introduced and *f*_eff_ is the mean atomic number of protein atoms (∼6.7; Crick & Magdoff, 1956 ▸). Applying this formulation to FcγRIII, the expected change 〈Δ*I*〉/*I* for both a mercury and a platinum derivative is ∼0.45 (assuming two heavy atoms per monomer in each case).

**Figure 6:**
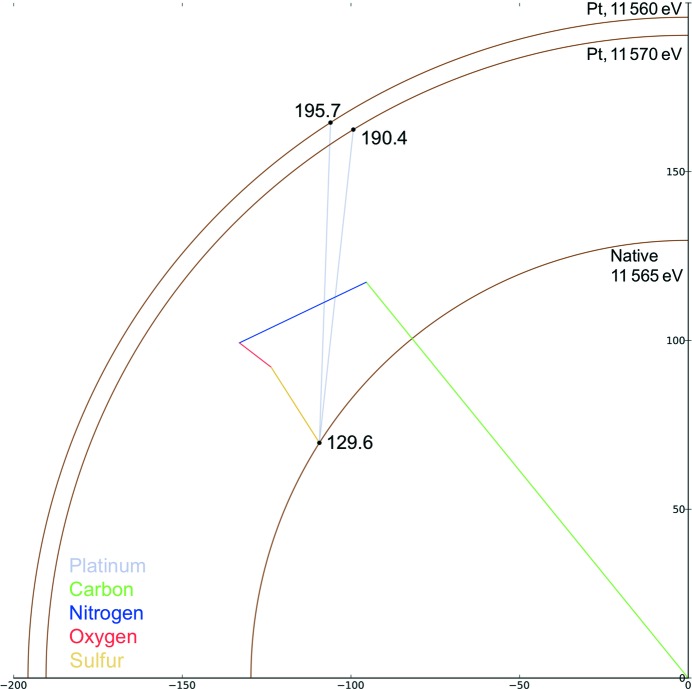
Argand diagram showing the change of reflection (17, 7, 6) for the platinum derivative of FcγRIII above and below the Pt *L*_III_ edge. The intensity increase with respect to the native is 128% (Pt derivative at 11.56 keV) and 116% (11.57 keV). The intensity change between the platinum derivatives above and below the *L*_III_ edge is 5.7%.

The Argand diagram in Fig. 6 ▸ shows how the addition of a single Pt atom to FcγRIII affects reflection (17, 7, 6). Just below the Pt *L*_III_ edge (11.56 keV; Pt *L*_III_ edge at 11.568 keV) the addition of platinum results in an intensity increase of 88% in comparison to the native. In the case of the derivative, changing the energy of the incident X-rays to just above the *L*_III_ edge changes the intensity. Increasing the energy to 11.57 keV decreases the intensity of reflection (17, 7, 6) by 6.1%. While the intensity change of 88% for reflection (17, 7, 6) is large in comparison to that of 3% resulting from disulfide-bond breakage, as in the case of disulfide breakage illustrated in Fig. 4 ▸, the addition of heavy atoms changes different reflections by different amounts. For the subset of reflections shown in Fig. 4 ▸ the addition of platinum causes a change of between −55 and +137% in the structure-factor intensity (the intensities of the reflections both increase and decrease), in comparison to a change of between −5.8 and +56% in the case of disulfide-bond damage. The median change in the intensity of a reflection owing to the introduction of platinum is 49% (based on a set of 10 000 reflections), closely matching the change predicted by the Crick and Magdoff formulation above. During structure solution it can be difficult to determine whether a change is owing to derivatization or damage, particularly if Friedel pairs are recorded at different doses during data collection.

## 7. How non-isomorphism affects structure factors   

Non-isomorphism between crystals can arise for a number of reasons. These may originate from variations in crystallization conditions, (de)hydration or the cryocooling process in addition to the main topics of this manuscript: radiation damage and derivatization. There are two dominant sources of non-isomorphism: changes in unit-cell parameters or changes in the relative positions of protein molecules. Crick & Magdoff (1956 ▸) showed that a relatively minor change in unit-cell parameters of 0.5%, or a shift of a protein molecule by 0.1 Å, will cause an average change in intensity of >15%. If non-isomorphism is introduced by derivatization, the expected intensity change owing to anomalous scattering (2) must therefore be somewhat greater than this if experimental phasing is to succeed. Non-isomorphism must also be considered for a single crystal type or derivative, as it is frequently not possible to obtain sufficient data from a single crystal, and small changes in the derivatization process, crystal handling or cryocooling may induce crystal-to-crystal variation. Radiation damage can also cause non-isomorphism to be introduced during the experiment, although the resulting size and rate of change of unit-cell parameters can be unpredictable, even for a particular crystal form (Murray & Garman, 2002 ▸; Ravelli *et al.*, 2002 ▸).

**Figure 7:**
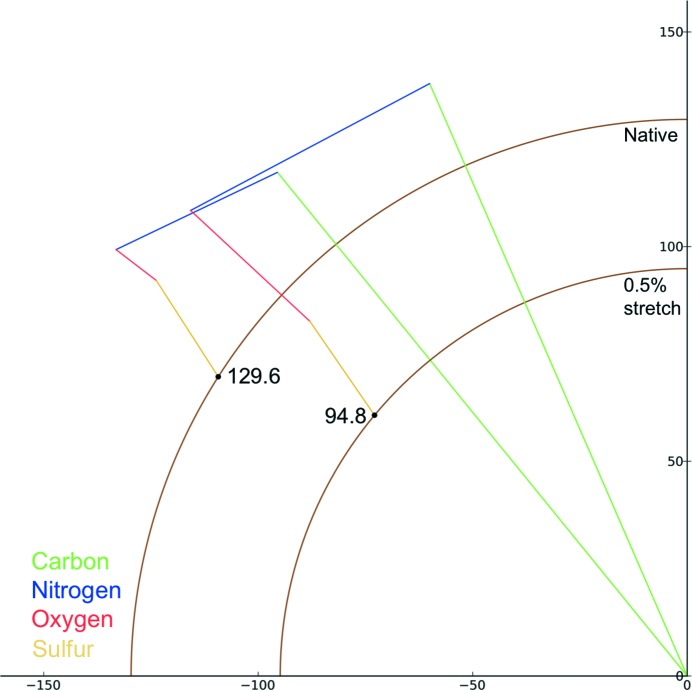
Change in structure factor (17, 7, 6) when the unit-cell parameters of FcγRIII are increased by 0.5% as detailed in the text.

Fig. 7 ▸ shows the change in reflection (17, 7, 6) resulting from a 0.5% increase in unit-cell parameters and a 0.15 Å shift of FcγRIII, with the intensity decreasing by 46%. These changes are intended to simulate a ‘breathing motion’ (*i.e.* a movement in which the unit-cell parameters change and simultaneously the molecules suffer a pure translation (without rotation) such that a point very approximately at the centre of gravity of FcγRIII maintains the same fractional coordinates in the unit cell). It is possible that crystal-to-crystal variation such as this may occur when heavy-atom soak times or the cryocooling protocol vary. A decrease in intensity might be expected when the unit cell is expanded as the X-ray beam passes through fewer unit cells, but as in the cases of site-specific damage and derivatization, non-isomorphism can cause the intensity of reflections to both increase and decrease and the magnitude of the change is not the same for all reflections. For the subset of reflections shown in Fig. 4 ▸, the above variations in the unit cell result in intensity changes of −46% (17, 7, 6), −12% (10, 33, 5), +14% (13, 12, −11) and +22% (12, 12, 12). In terms of an overall trend, the median intensity change in a set of 10 000 reflections is 30%.

**Figure 8:**
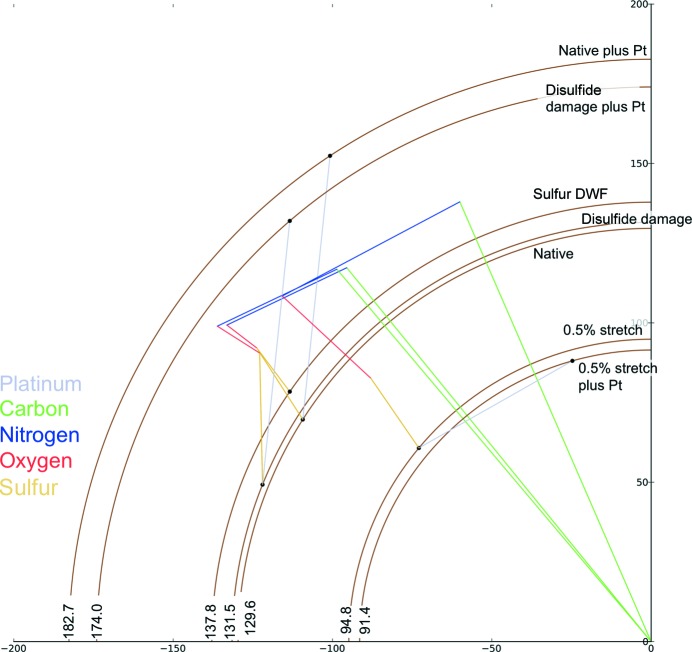
Comparison of all possible amplitudes of reflection (17, 7, 6) owing to the changes shown in previous figures. Included are native, platinum derivative, disulfide damage, ‘hot’ S atoms and an expanded unit cell. Combinations of the above are also shown.

Fig. 8 ▸ shows the variation in reflection (17, 7, 6) taking into account all of the above effects, *i.e.* disulfide stretching and increasing the *B* factor, changes in the unit-cell volume and the addition of heavy atoms. Taking into account the different possible combinations of these, there are many possible structure factors, seven of which are shown. The large possible number of intensities and variation with respect to the native illustrate the care that must be taken when collecting and interpreting diffraction data.

## 8. Summary   

§5, §6 and §7 illustrate how radiation damage, derivatization and non-isomorphism can affect structure factors. Radiation damage in macromolecular crystallography results in global effects, such as a decrease in diffracting power, on dose scales of the order of tens of megagrays and site-specific damage, which occurs much faster, on dose scales of tens of kilograys. Both of these dose scales can easily be, and routinely are, surpassed during data collection at synchrotron sources, with the result that both types of damage can be considered to be an inevitable part of structure determination. Site-specific damage results in different rates of decay for individual reflections and we have shown that the size of these changes in intensity is comparable to those introduced by derivatization. The primary way in which radiation damage can be reduced is through reduction of the dose absorbed by a crystal during data collection. This can be achieved most simply by decreasing the exposure time/increasing the attenuation of the X-ray beam, but also by increasing the beam size to match that of the crystal (if applicable), translating the crystal through the beam during data collection or through the merging of low-dose partial data sets from multiple crystals. Prior to data collection, efficient backsoaking of derivatives removes heavy atoms which contribute greatly to the absorbed dose but do not add anomalous signal. Non-isomorphism can be minimized through consistent crystal handling and treatment during, for example, the mounting or cryocooling processes. If multiple crystals are used then software such as *BLEND* (Foadi *et al.*, 2013 ▸) can be used to group crystals into clusters with similar unit-cell parameters.

Radiation damage, non-isomorphism and derivatization are intrinsically linked in macromolecular crystallography. These processes do not occur independently and the observed intensities will differ from those resulting from an ‘undamaged native’ crystal owing to some combination of all of the above. The optimal experimental strategy must therefore seek to minimize the contribution of radiation damage and non-isomorphism to maximize the chance that the differences in intensity recorded represent changes owing to derivatization.

## Figures and Tables

**Table 1:** Typical doses for a macromolecular experiment at a third-generation synchrotron source Doses are compared for two proteins using the parameters of two beamlines at Diamond Light Source: I03 and I24 (http://www.diamond.ac.uk/Beamlines/Mx/).

	FcγRIII		ABC transporter	
Energy (keV)	12.8	12.8	12.8	12.8
Beam size (µm)	10 × 10	80 × 20	10 × 10	80 × 20
Flux (photons s^−1^)	1.5 × 10^12^	1.7 × 10^12^	1.5 × 10^12^	1.7 × 10^12^
Dose rate (MGy s^−1^)	5.27	0.37	5.50	0.39

**Table 2:** Comparison of absorbed doses for derivatives of FcγRIII All doses are calculated at 14.9 keV (just above the Hg *L*_I_ edge) using the I24 beam parameters given in Table 1 ▸ (beam size 10 × 10 µm, 1.5 × 10^12^ photons s^−1^). Changes as a function of energy are illustrated in Fig. 1 ▸. Time to *I*_95_ is the estimated time taken reduce the diffracting power by 5% based on the assumption that an absorbed dose of 43 MGy reduces the diffracting power by 50% (Owen *et al.*, 2006 ▸).

	Native	K_2_PtCl_4_	KAuCl_4_	HgCl_2_	K_2_PtCl_4_ with no backsoak
No. of heavy atoms per monomer	—	2	2	2	2 + 250 m*M*
Absorption coefficient (mm^−1^)	0.12	0.35	0.35	0.33	0.76
Dose rate (MGy s^−1^)	3.74	9.17	9.09	8.61	18.8
Time to reach 30 MGy (s)	8.0	3.7	3.3	3.48	1.60
Time to *I*_95_ (s)	1.14	0.47	0.47	0.50	0.23
